# Hyper-fractionated radiotherapy as a bridging strategy to enhance CAR-T efficacy by regulating T-cell co-stimulatory molecules in relapsed/refractory diffuse large B-cell lymphoma

**DOI:** 10.3389/fimmu.2024.1481080

**Published:** 2024-12-02

**Authors:** Jing Ruan, Daobin Zhou, Yan Zhang, Danqing Zhao, Chong Wei, Ke Hu, Fuquan Zhang, Xiaorong Hou, Wei Zhang

**Affiliations:** ^1^ Department of Hematology, Peking Union Medical College Hospital, Beijing, China; ^2^ Department of Radiotherapy, Peking Union Medical College Hospital, Beijing, China

**Keywords:** hyper-fractionated radiotherapy, DLBCL, CAR-T, bridging therapy, T-cell co-stimulatory molecules

## Abstract

**Background:**

Bridging therapy can prevent patients from disease progression while waiting for CAR-T cell preparation. Hyper-fractionated radiotherapy can achieve an effective target dose within a short period, minimize radiation damage, and may modify immune environment compared to conventional radiotherapy.

**Aims:**

This study aims to investigate the efficacy and safety of bridging hyper-fractionated radiotherapy in combination with CAR-T therapy for relapsed/refractory diffuse large B-cell lymphoma. The potential mechanisms were explored.

**Methods:**

This is a prospective pilot study. After T-cell collection, the patients underwent hyper-fractionated radiotherapy at lesion sites with 1.5 Gy twice daily for 10 days before CAR-T cell infusion. Peripheral blood immune cell subsets and quantitative serum proteomics were assessed before radiotherapy and after radiotherapy before CAR-T cell infusion.

**Results:**

A total of 13 patients have been enrolled. The median follow-up time was 6 (3–24) months after CAR-T infusion. At 3-month follow-up, 9/13(69%) patients had CR, 1/13(8%) patient had PR, 1/13(8%) patient remained SD, and 2/13(15%) patients died of disease progression. The local recurrence rate was 1/13(8%). Seven patients have been followed up for more than 6 months, and they remain in CR. The median PFS and OS were not reached. No grade 3–4 CRS or ICANS were reported. After hyper-fractionated radiotherapy, peripheral PD1+CD8+T/T ratio significantly decreased while quantitative serum proteomics profiling showed a decrease in sCD28.

**Conclusion:**

Hyper-fractionated radiotherapy can rapidly control tumor progression sites without delaying the infusion time. This approach can improve the ORR and does not increase the incidence of CRS and ICANS. The mechanism may be related to the regulation of T-cell co-stimulatory molecules, which demands further exploration.

## Highlights

- Hyper-fractionated radiotherapy can rapidly control tumor, potentially improve the outcome of CAR T therapy, and does not appear to increase the incidence of CRS and ICANS.
- T-cell co-stimulatory molecules were modified by hyper-fractionated radiotherapy to enhance CAR-T efficacy.

## Background

Patients with relapsed/refractory diffuse large B-cell lymphoma have poor overall survival with few treatment options. CAR-T therapy is a breakthrough in helping these patients to achieve long-term remission.

However, the preparation of CAR-T cell usually takes approximately 4 weeks. To prevent disease progression that would affect CAR-T cell infusion, bridging therapy is needed in most patients (1). The current approach for bridging therapy mainly involves conventional chemotherapy, while patients with relapsed/refractory diffuse large B-cell lymphoma often develop resistance. Some patients may even have progressively large mass that compress important organs, resulting in liver and kidney dysfunction, pain, and even paralysis.

Local radiotherapy can effectively and significantly reduce tumors and improve symptoms in these patients. Cases have reported the effectiveness and safety of radiotherapy bridging CAR-T therapy in refractory relapsed lymphoma (2, 3). Recently, several retrospective studies have confirmed that radiotherapy bridging can be safely and effectively used even in advanced stage and high-risk disease, while whether it brings superior outcome than systematic treatment remains controversial (4–7). Furthermore, patients who received radiotherapy before CAR-T seemed to have lower incidence of life-threatening toxicities including cytokine release syndrome (CRS) and immune effector cell-associated neurotoxicity syndrome (ICANS) (8). The previously reported cases in the literature mostly employ conventional fractionation as bridging treatment. In fact, hyper-fractionated radiotherapy can achieve an effective target dose within a shorter period and minimize radiation damage compared to conventional radiotherapy. In addition, studies in mice models proved that low-dose radiotherapy may promote the recruitment of CAR-T cells to tumors and enhance CAR-T cell cytotoxicity. The tumor microenvironment may also be modified to improve immune escape (9, 10).

Therefore, in this pilot study, we aimed to prospectively investigate the efficacy and safety of bridging hyper-fractionated radiotherapy in combination with CAR-T therapy for relapsed/refractory diffuse large B-cell lymphoma. The potential mechanisms would also be explored (NCT05514327).

## Methods

### Patient population

This is a pilot study. We prospectively enrolled relapsed/refractory diffuse large B-cell lymphoma patients with measurable lesions in Peking Union Medical College Hospital between 2022 and 2024. Written informed consent was signed by the patients. The study was approved by the ethics committee of Peking Union Medical College Hospital and was conducted in accordance with the Declaration of Helsinki.

### Treatment and follow-up

After T-cell collection, the patients underwent hyper-fractionated radiotherapy at the sites of maximum lesion size (or highest SUV), rapidly progressing sites, and sites with compressive symptoms (total radiation sites ≤3). The radiotherapy regimen consisted of 1.5 Gy twice daily for 10 days. CAR-T cell infusion was performed 1–2 weeks after the completion of radiotherapy. Adverse reactions at different time points after radiotherapy and CAR-T cell infusion were monitored and analyzed. CRS and ICANS were documented by primary care physicians based on consensus guidelines from the American Society of Transplantation and Cellular Therapy for Cytokine Release Syndrome and Neurologic Toxicity Associated with Immune Effector Cells (11). Radiation-related adverse events were graded using Common Terminology Criteria for Adverse Events version 5.0. PET/CT was scheduled at 1 month, 3 months, 6 months, 1 year, and 2 years to evaluate the disease according to the Lugano criteria. Peripheral blood immune cell subsets were assessed by flow cytometry before and after radiotherapy (12, 13). Plasma was also obtained and analyzed using an Olink proteomics Target-96 immuno-oncology-panel that included multiple markers of angiogenesis and proliferation (14, 15).

### Statistical analysis

Descriptive variables were displayed as percent of all patients with available data, while continuous variables were expressed as mean ± SEM. Quantitative data were compared by two-sample (unpaired Student’s) two-tailed t-test assuming equal variance, while categorical data were compared using the Chi-square test. Comparisons among groups were calculated by paired-samples t-test. Progression-free survival (PFS) and overall survival (OS) were analyzed using Kaplan–Meier survival analysis. A two-sided p-value of <0.05 was considered statistically significant. All the statistical tests were performed using SPSS 21.0 statistical software.

## Results

### Patient characteristics

From 2022 to 2024, 13 relapsed/refractory diffuse large B-cell lymphoma (DLBCL) patients have been prospectively enrolled in our study. Baseline clinicopathological features are listed in **Table 1**. The median age was 52 years old (27–66 years), and 69% of them were men. Four (31%) were GCB type, five (38%) were double expressor, one (8%) was double hit, and one (8%) was transformed from marginal zone B-cell lymphoma. Seven (54%) patients were stage IV at diagnosis according to Ann Arbor stages, and the international prognostic index (IPI) scores at diagnosis were commonly 2–3 (54%). Four (31%) patients had bulky disease before radiotherapy, which was defined by one or more lesions ≥7.5 cm in greatest dimension. Six (46%) patients were primary refractory. The median lines of therapy prior to CAR-T were three (two to six lines), and two (15%) of them had undergone previous autologous stem cell transplantation.

**Table 1:** Basic characteristics of the patients enrolled in our study.

Basic characteristics	Value
Age at BRT, year, median (range)	52 (27–66)
Sex, n(%)	
Male	9 (69%)
Female	4(31%)
Pathology at diagnosis, n(%)	
DLBCL	12 (92%)
Transformed marginal zone B-cell lymphoma	1 (8%)
Germinal center type	4 (31%)
Double expressor	5 (38%)
Double/triple hit	1 (8%)
Ann Arbor stage at diagnosis, n(%)	
IEA	2 (15%)
II	4 (31%)
IV	7 (54%)
International prognostic index score at diagnosis, n(%)	
Low (0–1)	5 (38%)
Intermediate (2–3)	7 (54%)
High (4–5)	1 (8%)
Bulky disease (≥7.5 cm in greatest dimension)	4 (31%)
Primary refractory disease, n (%)	6 (46%)
Lines of therapy prior to CAR-T, n median (range)	3 (2–6)
Prior autologous stem cell transplantation, n (%)	2 (15%)
Radiotherapy field	
Comprehensive	3 (23%)
Focal	6 (46%)
WBRT	4 (31%)
CAR-T cell product	
Relmacabtagene autoleucel	8 (62%)
Axicabtagene ciloleucel	5 (38%)

### Bridging therapy characteristics

Four (31%) patients received whole brain radiotherapy, among whom one was primary central nervous system lymphoma (PCNSL) and three patients were systemic DLBCL. The other radiotherapy treated anatomic sites including the neck (n=2), mediastinum (n=2), breast (n=1), abdomen (n=3), and pelvis (n=1). Hyper-fractionated radiotherapy was prescribed for all patients; in detail, the total dose was 30 Gy for all patients, and the regimen was 1.5 Gy twice a day in continuous 10 days. Prescription coverage of 100% of 95% of the planned gross tumor volume (PGTV) was performed. Intensity-modulated radiation therapy (IMRT) was performed for all patients. As for the radiotherapy field, three (23%) patients had comprehensive radiotherapy, which was defined as treating all active sites of disease seen on PET/CT; six (46%) patients had focal radiotherapy, which was defined in cases where not all disease was treated; and four (31%) patients received whole brain radiotherapy (WBRT) (**Supplementary Table S1**).

Five (38%) patients also received systemic therapy during the bridging period. Two patients received orelabrutinib continuously to CAR-T cell infusion, while the dosage was reduced during radiotherapy. The other three patients were treated with VP16 and dexamethasone, oxaliplatin and dexamethasone, brentuximab vedotin, and tislelizumab, separately.

The time interval between the completion of radiotherapy and CAR-T cell infusion was 7–25 days (median, 12 days). All patients received fludarabine and cyclophosphamide lymphodepletion before CAR-T cell infusion. The CAR-T cell products were relmacabtagene autoleucel in eight (62%) patients and axicabtagene ciloleucel in five (38%) patients.

### Clinical outcomes

#### Clinical response and survival outcomes

**Figure 1:**
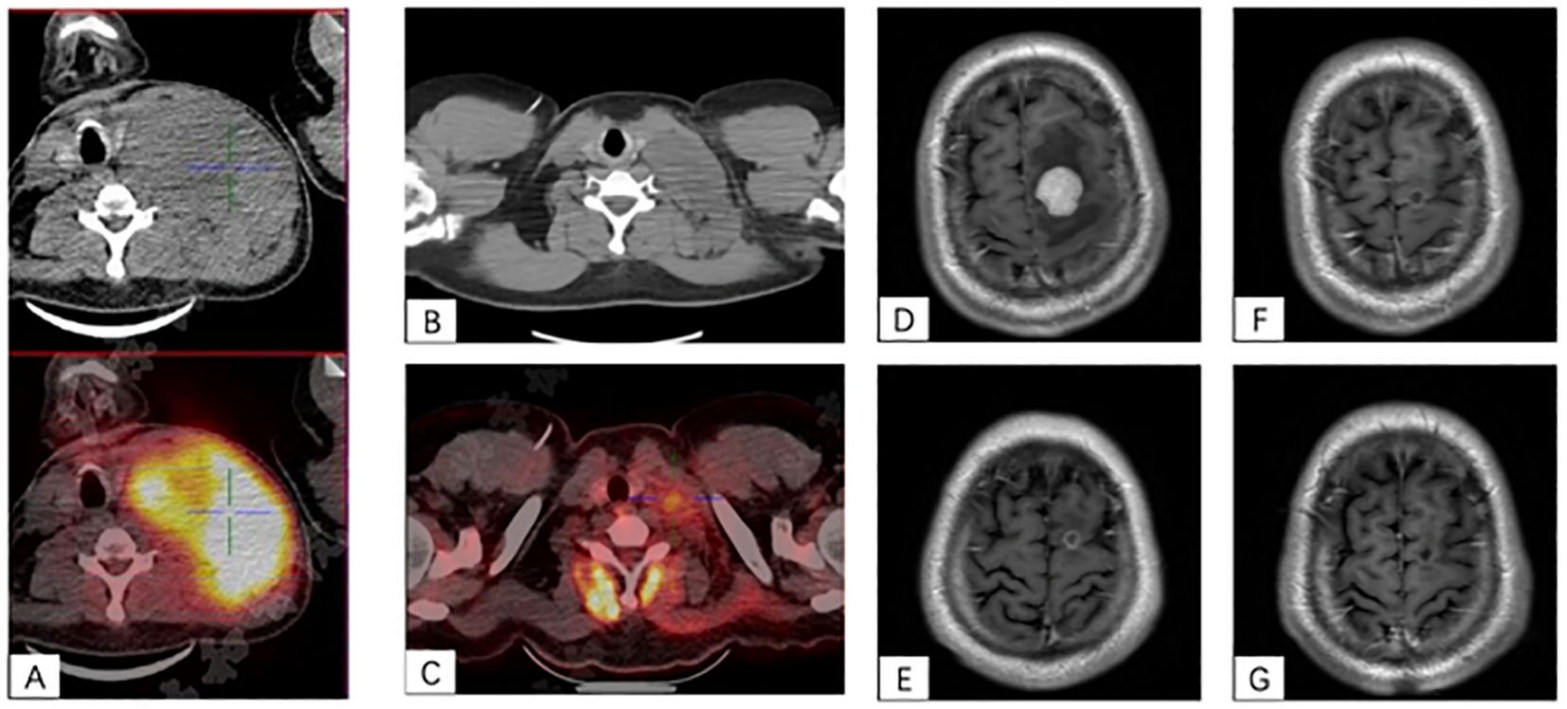
**(A–C)** A case of bulky left cervical disease. **(A)** Baseline PET/CT scan showed a 13.1 cm × 8.4 cm × 10.5 cm mass with SUVmax to be 20.5 at the left side of the neck and pushing the trachea to the right. **(B)** The mass quickly shrank to 6×2.6 cm after hyper-fractionated radiotherapy. **(C)** After CAR-T cell infusion at 1 month, PET/CT scan showed a 3.2 cm × 1.9 cm mass with SUVmax of 3.2. **(D–F)** A case of CNS lesion. **(D)** Baseline contrast-enhanced MRI showed a 2.5-cm enhanced nodule with peripheral edema in the left frontal lobe. **(E)** After radiotherapy, the lesion shrank to 0.75 cm with ring-like enhancement. **(F)** After CAR-T cell infusion at 1 month, the lesion was 0.5 cm with ring-like enhancement. **(G)** The lesion had no enhancement on the contrast-enhanced MRI at 3-month after CAR-T cell infusion.

All of them achieved partial response (PR) after the completion of hyper-fractionated radiotherapy (**Figure 1**) and successfully completed CAR-T cell infusion later. The detailed information is shown in **Supplementary Table S1**.

**Figure 2:**
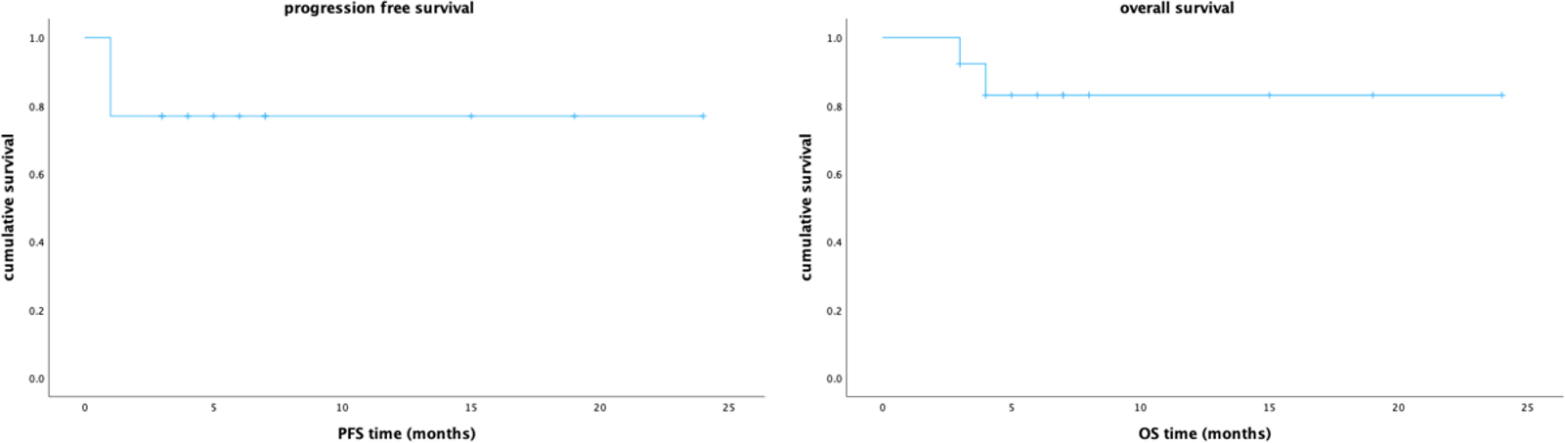
The Kaplan–Meier survival curves for progression free survival and overall survival of the 13 patients in our study.

Till submission, the median follow-up time was 6 (3–24) months after CAR-T infusion. At 1-month follow-up, 7/13 (54%) patients achieved CR, 2/13 (15%) achieved PR, 1/13 (8%) achieved SD, and 3/13 (23%) achieved PD; the ORR was 69%. At 3-month follow-up, 9/13(69%) patients had CR and 1/13 (8%) patient had PR, 1/13 (8%) patient remained SD, and 2/13 (15%) patients died of disease progression; the ORR was 77%. Seven patients have been followed up for more than 6 months, and they remain in CR. The median PFS and OS were not reached (**Figure 2**).

The local recurrence rate defined by disease progression inside the irradiated field was 1/13 (8%), and this patient was PCNSL who received WBRT. Two patients with PR at 1-month follow-up had residual lesions at the site of prior radiotherapy and received salvage radiotherapy at the same site; both achieved CR at 3-month follow-up. One patient had partial response in in-field radiotherapy site (abdominal disease), while bone lesions outside the radiotherapy field were progressed at 1-month follow-up after CAR-T cell infusion. He achieved PR at 3-month follow-up with salvage therapy of four cycles of tislelizumab and ibrutinib; CR was reached at 6-month follow-up with ibrutinib maintenance.

### Adverse effects

The adverse effects after radiotherapy and before CAR-T cell infusion were limited. One patient experienced cerebral edema and relieved after dexamethasone. One patient had aspiration pneumonia and recovered from antibiotics. Two patients had grade 4 neutropenia; one of them received orelabrutinib during radiotherapy, and the other one had systemic chemotherapy therapy before radiotherapy during the bridging period.

Following CAR-T cell infusion, CRS was observed in 10/13 (77%) patients, and 5/13 (38%) were grade 1, 5/13 (38%) were grade 2. No grade 3–4 CRS or ICANS were reported. Grade 3 neutropenia was reported in 2/13 (15%) patients, and grade 4 neutropenia was reported in 10/13 (77%) patients after CAR-T cell infusion. The median time from infusion to neutropenia was 3.5 days (−1 to 14 days), the median time for neutropenia recovery was 5.5 days (1–29 days), and 10/12 (83%) recovered within 1 week. One patient had bloodstream infection, and two patients had COVID-19 infection. Grade 1–3 thrombocytopenia was observed in 6/13 (46%) patients.

### Peripheral blood immune cell subsets and quantitative serum proteomics before and after hyper-fractionated radiotherapy

**Figure 3:**
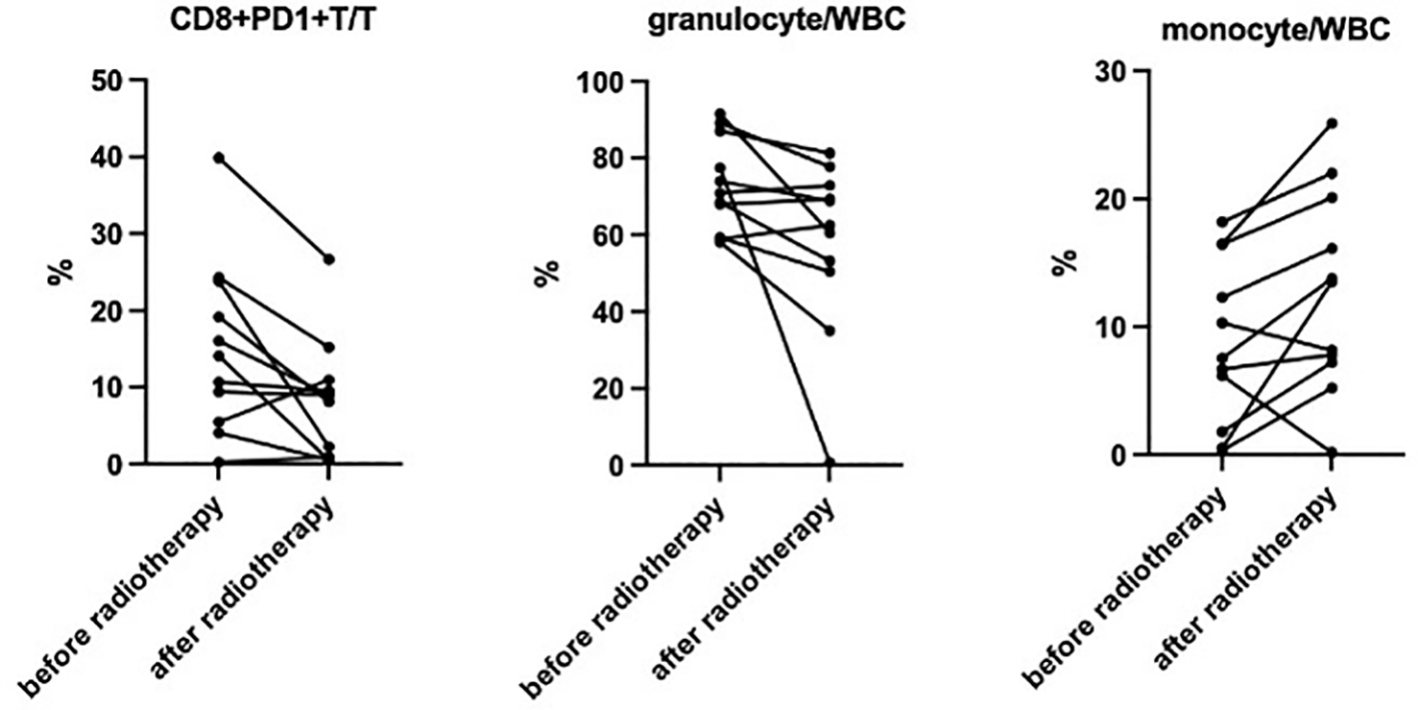
Significantly decreased CD8+PD1+T/T (15% vs. 8%, p=0.017), decreased granulocyte/WBC (73% vs. 58%, p=0.049), and increased monocyte/WBC (9% vs. 13%, p=0.029) ratio were observed after hyper-fractionated radiotherapy.

**Figure 4:**
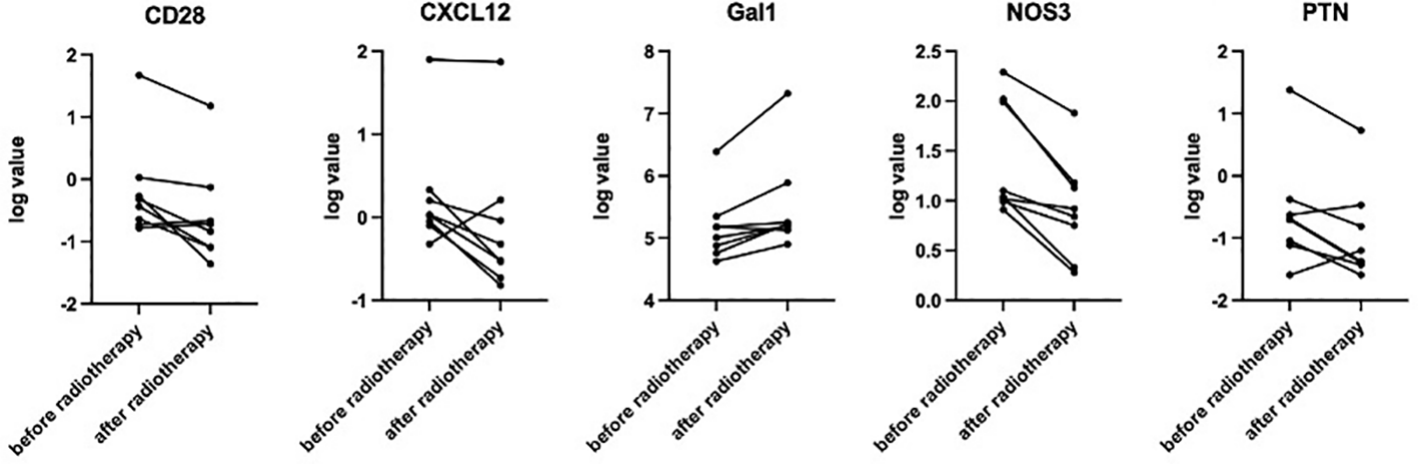
Paired t-test showed significantly decreased sCD28 (p=0.016), CXCL12 (p=0.013), NOS3 (p=0.003), and PTN (p=0.034) and increased Gal1 (p=0.009) after hyper-fractionated radiotherapy.

The percentage and absolute number of peripheral blood immune cell subsets before and after hyper-fractionated radiotherapy were compared (**Supplementary Tables S2, S3**). Preliminary analysis showed that after hyper-fractionated radiotherapy, peripheral PD1+CD8+T/T ratio significantly decreased (15% vs. 8%, p=0.017) (**Figure 3**), while the absolute number of PD1+CD8+T tended to be decreased (120×10^6^/L vs. 46×10^6^/L, p=0.09). Peripheral monocyte/WBC ratio also increased (9% vs. 13%, p=0.029) and granulocyte/WBC ratio decreased (73% vs. 58%, p=0.049). There were no differences between percentage of naïve T cell, memory T cell, effector T cell, Treg cell, TEMRA cell, Tim3+ T cell, NK cell, and dendritic cell. Furthermore, the absolute numbers of total Treg (15.7×10^6^/L vs. 10.9×10^6^/L, p=0.027) and CD45 RA-Treg (11.5×10^6^/L vs. 8.2×10^6^/L, p=0.025) subsets were significantly decreased, while the percentage of these subsets remained unchanged after radiotherapy. Quantitative serum proteomics profiling showed a decrease in sCD28 (p=0.016). CXCL12 (p=0.013), NOS3 (p=0.003), and PTN (p=0.034) also significantly decreased and Gal1 (p=0.009) (**Figure 4**) increased after hyper-fractionated radiotherapy. GO enrichment analysis showed that the differentially expressed proteins were mainly involved in immune cell chemotaxis and signal transduction.

## Discussion

Here, we reported the first prospective study about the efficacy and safety of bridging CAR-T with hyper-fractionated radiotherapy in relapsed/refractory diffuse large B-cell lymphoma.

The integration of radiotherapy as a bridging strategy prior to CAR-T cell therapy is an emerging approach being explored in the management of lymphoma. Importantly, the radiotherapy did not appear to significantly impair CAR-T expansion or persistence. Within the limits of the small cohorts retrieved, radiotherapy seems a superior option compared with systemic treatment as a “bridge” to CAR-T and could as well reduce severe complications rates (4, 16). In the largest multi-center retrospective cohort from the UK, 169/717(24%) patients received bridging radiotherapy before CAR-T cell infusion. The best ORR/CR rates by single-modality radiotherapy, combined modality treatment (CMT), and systemic bridging were 82.1%/62.5%, 81.8%/63.6%, and 74%/55.9%, respectively. Furthermore, radiotherapy-bridged patients had favorable outcomes with 1-year PFS of 56% for single modality and 47% for CMT, while 1-year PFS for systemic bridging was 43%. In a study from China, 29/60 patients received radiotherapy before CAR T-cell infusion. Compared to the 31/60 patients with only bridging chemotherapy, the radiotherapy bridging group achieved a higher ORR at day 30 (82.8% vs. 45.2%), better 1-year PFS (46.9% vs. 22.6%) while reduced CRS of grade≥3; patients with bulky disease even had better 1-year OS (17). However, another multi-institutional study showed that the patients with radiotherapy bridging had lower median OS compared to the CMT and systemic bridging group despite comparable baseline characteristics (5). In our prospectively pilot study, CR at 1 month and at 3 months was 54% and 69%, respectively, while the ORR was 60% and 77%, respectively. A total of 54% patients who have been followed up for more than 6 months remained CR. The ORR tended to be higher than in patients without bridging therapy and was comparable to patients who received conventional radiotherapy, while the rate of CR at more than 3 months tended to be higher compared to historical records (1, 18). The median PFS and OS have not been reached with the median follow-up time of 6 (3–24) months in our report.

Debulking tumor burden before infusion is important to improve CAR-T therapy outcome and reduce side effects. Bridging radiation therapy could effectively cytoreduce high-risk relapsed/refractory aggressive B-cell lymphoma after multi-lines therapy including tumor diameter, SUV, and serum LDH, all predictors of poor post-CAR-T therapy outcomes (18). Currently, no consensus exists for dose/fractionation for the purposes of bridging RT, and an interesting pilot trial by Ababneh is ongoing (5). Our study innovatively utilized hyper-fractionated radiotherapy where the total radiation dose is delivered in more frequency and lower single doses compared to conventional fractionation. Therefore, an effective target dose could be achieved within a shorter period to rapidly effectively shrink the tumor load without delay or affect CAR-T cell infusion and could minimize radiation damage.

In addition, low-lose radiation may have a better synergistic effect with CAR-T therapy due to its potential role in tumor recruiting and immune modulating. According to DeSelm, low-dose radiation conditioning enables CAR-T cells to mitigate antigen escape in a TRAIL-dependent manner (19). Furthermore, radiation-induced IFN-gamma production within the tumor microenvironment may help CAR-T cell infiltration and tumor cell target recognition in glioblastoma mice model (20, 21). In our study, we found that peripheral PD1+CD8+T and sCD28 decreased after hyper-fractionated radiotherapy. CD28 and PD-1 are two important co-stimulatory molecules that play a critical role in T-cell activation and regulation of immune responses. CD28 binds to the B7 molecules (including CD80 and CD86) on the surface of antigen-presenting cells, providing the second signal to promote T-cell activation and proliferation. PD-1, on the other hand, is a negative regulatory molecule mainly expressed on activated immune cells such as T cells, and its binding to PD-L1 suppresses T-cell activation and function. After hyper-fractionated radiotherapy, the level of serum soluble CD28 (sCD28) decreased, and since previous studies have shown a negative correlation between serum sCD28 and the level of CD28 on the T-cell membrane surface (22), we speculate that hyper-fractionated radiotherapy can increase the level of CD28 on the surface of T cells in DLBCL patients, thereby enhancing the anti-tumor effect. However, it is a pity that we did not prospectively perform flow cytometry to assess CD28 expression on T cells. On the other hand, PD1+CD8+T cells play an exhaustive role in the tumor microenvironment. Studies have proved that decreased peripheral PD1+CD8+ T cells after immunotherapy may indicate better outcome (23), and a higher proportion of PD1+CD8+T in the serum corresponded to shortened PFS in lung cancer (24). Therefore, we hypothesize that hyper-fractionated radiotherapy also helps improve the tumor immune microenvironment by reducing the percentage of PD1+CD8+ T cells. Further direct studies should be conducted to explore the potential mechanisms. Notably, three patients evaluated to be PR/PD at 1 month finally reached CR at 3 months or 6 months after salvage therapy including complementary radiotherapy or PD-1 blockade. According to Ababneh and Saifi, patients with limited post-CART disease who received salvage comprehensive RT had better overall survival and freedom from subsequent progression (25, 26). Radiotherapy or PD-1 may help priming the tumor microenvironment and enhance tumor antigen presentation (25, 27, 28) even after CAR-T cell infusion, and several related clinical trials have been ongoing. Another interesting finding is the decreased number of Treg cells after hyper-fractionated radiotherapy, especially the CD45RA− Treg cells. CD45RA− Treg cells are mainly effector Treg and effector memory Treg cells (29). Studies have shown that CD45RA− Treg cell subsets have immunosuppressive properties, and the accumulation of CD45RA− Treg in tumor microenvironment correlated with tumor progression and poor prognosis in solid tumors (30–32). Furthermore, the infiltration of CD4+ Treg cells could decrease the efficacy of CAR-T therapy by CTLA-4 expression and IL-2 production. Therefore, hyper-fractionated radiotherapy may help enhance the efficacy of CAR-T by decreasing CD45RA− Treg cells.

There are some important safety considerations that need to be carefully monitored when combining radiotherapy and CAR-T cell therapy. First of all, since both radiotherapy and CAR-T cell therapy can cause myelosuppression, cytopenia, and infections, it has drawn attention. In our study, although grade 3–4 neutropenia was common, the duration of neutropenia was less than 7 days for most patients, and no fatal infection was recorded. Still, a close monitoring of blood counts and infection prevention are suggested especially for patients with combined bridging therapy other than radiotherapy or for special anatomy radiation field including whole brain radiotherapy. Furthermore, CAR-T cell therapy can trigger severe irAEs, such as CRS and ICANS; the addition of hyper-fractionated radiotherapy may potentially enhance immune response. However, no grade 3–4 CRS or ICANS were reported in this study. The addition of radiotherapy or even hyper-fractionated radiotherapy in turn reduces the incidence or severity of CRS and ICANS, which may be due to reduced tumor burden (33). In addition, the combination of radiotherapy and CAR-T cell therapy may theoretically increase the risk of developing secondary cancers, especially given the potential for radiotherapy-induced genomic instability. Long-term follow-up and surveillance will be crucial to monitor for this potential complication.

This study has several limitations. Most notably, the sample size is relatively small, which also limits subgroup analyses, and the follow-up duration was relatively short to have long-term efficacy and survival data. We would continue to enroll more patients and update the data with longer follow-up time. In addition, 38% patients also received other systemic therapy in addition to hyper-fractionated radiotherapy during the bridging period, which may affect both outcomes and toxicity, although it may reflect real-world practice patterns. Moreover, we did not perform a PET/CT evaluation before CAR-T cell infusion to evaluate the effect of hyper-fractionated radiotherapy to help provide more interesting information.

## Conclusion

In conclusion, bridging hyper-fractionated radiotherapy with CAR-T therapy is safe and effective for patients with relapsed/refractory diffuse large B-cell lymphoma. Ultra-hyper-fractionated radiotherapy can rapidly control tumor progression sites without delaying the infusion time. This approach can improve the outcome and does not increase the incidence of CRS and ICANS. Further follow-up is needed to assess long-term efficacy and survival. The mechanism may be related to the regulation of T-cell co-stimulatory molecules by hyper-fractionated radiotherapy, which demands further exploration.

## Data Availability

The original contributions presented in the study are included in the article/**Supplementary Material**. Further inquiries can be directed to the corresponding authors.
