# Supplementary material for: Hyper-fractionated radiotherapy as a bridging strategy to enhance CAR-T efficacy by regulating T-cell co-stimulatory molecules in relapsed/refractory diffuse large B-cell lymphoma
Source: Front Immunol. 2024 Dec 2;15:1481080. doi: 10.3389/fimmu.2024.1481080 (PMC11646978; doi:10.3389/fimmu.2024.1481080)
Supplement: Supplementary file 1 [file Table1.docx]

**Supplementary Table 1: detailed radiotherapy information of the patients in our study.**

| **No.** | **diagnosis** | **Lesions before RT** | **Target lesion of RT** | **RT field** | **RT technique** | **Dosage/ fraction** | **Baseline target lesion size(cm)^*^** | **Target lesion size after RT(cm)^**^** | **Evaluation method after RT and before CAR-T** | **RT-related toxicity** |
| --- | --- | --- | --- | --- | --- | --- | --- | --- | --- | --- |
| 1 | DLBCL | Brain | Brain | WBRT | IMRT | 30Gy/20 | 3 | 1 | MRI | Grade IV neutropenia |
| 2 | DLBCL | Brain, stomach | Brain | WBRT | IMRT | 30Gy/20 | 1.5 | 0.74 | MRI | Grade IV neutropenia |
| 3 | DLBCL | Pharyngeal and cervical lymph nodes | Pharyngeal and cervical lymph nodes | comprehensive | IMRT | 30Gy/20 | 3.5 | 1 | CT | None |
| 4 | DLBCL | Bones, right kidney, Lymph nodes adjacent to the abdominal aorta and inferior vena cava | Right kidney, Lymph nodes adjacent to the abdominal aorta and inferior vena cava | focal | IMRT | 30Gy/20 | 9 | 6.9 | CT | None |
| 5 | DLBCL | Left cervical and anterior mediastinal mass, left axillary lymph nodes | Left cervical and anterior mediastinal mass | focal | IMRT | 30Gy/20 | 13.1 | 6 | CT | None |
| 6 | PCNSL | Brain | Brain | WBRT | IMRT | 30Gy/20 | 3.5 | 2.3 | MRI | Pneumonia |
| 7 | DLBCL | Left axillary lymph nodes, Lymph nodes adjacent to the abdominal aorta and hepatic hilus | Lymph nodes adjacent to the abdominal aorta and hepatic hilus | focal | IMRT | 30Gy/20 | 1 | 0.5 | CT | None |
| 8 | DLBCL | Lymph nodes posterior to pancreatic head | Lymph nodes posterior to pancreatic head | comprehensive | IMRT | 30Gy/20 | 3.3 | 1 | CT | None |
| 9 | DLBCL | Right nasopharyngeal lymph nodes, right cervical mass | Right cervical mass | focal | IMRT | 30Gy/20 | 10 | 5 | ultrasound | None |
| 10 | DLBCL | Mediastinal mass, pericardium, pleura | Mediastinal mass | focal | IMRT | 30Gy/20 | 11 | 7.3 | CT | Grade III neutropenia |
| 11 | DLBCL | Left pelvic side wall | Left pelvic side wall | focal | IMRT | 30Gy/20 | 4.2 | 3.5 | CT | None |
| 12 | DLBCL | Left breast | Left breast | comprehensive | IMRT | 30Gy/20 | 4.1  SUVmax16 | 3.1  SUVmax4 | PET/CT | None |
| 13 | tMZL | Brain | Brain | WBRT | IMRT | 30Gy/20 | 2.5 | 0.75 | MRI | Brain edema |

Abbreviations: RT, radiotherapy; DLBCL, diffuse large b cell lymphoma; WBRT, whole brain radiotherapy; IMRT, intensity-modulated radiation therapy; tMZL, transformed marginal zone B cell lymphoma.

* Greatest diameter of the target lesion before irradiation

** Greatest diameter of the target lesion after irradiation
