# Supplementary material for: Hyper-fractionated radiotherapy as a bridging strategy to enhance CAR-T efficacy by regulating T-cell co-stimulatory molecules in relapsed/refractory diffuse large B-cell lymphoma
Source: Front Immunol. 2024 Dec 2;15:1481080. doi: 10.3389/fimmu.2024.1481080 (PMC11646978; doi:10.3389/fimmu.2024.1481080)
Supplement: Supplementary file 2 [file Table2.docx]

**Supplementary Table 2: comparison of the peripheral blood immune cell subsets percentage before and after hyper-fractionated radiotherapy analyzed by pared-T test.**

| **cell subsets** | **Phenotype** | **before radiotherapy (%)** | **after radiotherapy (%)** | **SD of average changes** | **P value** |
| --- | --- | --- | --- | --- | --- |
| T/Lym | CD3+/Lym | 83.6864 | 83.7964 | 6.27222 | 0.986 |
| CD4+T/Lym | CD3+CD4+/Lym | 34.0191 | 38.2373 | 6.3404 | 0.521 |
| CD8+T/Lym | CD3+CD8+/Lym | 42.3945 | 39.5618 | 4.7084 | 0.561 |
| CD4+T/CD8+T | CD4/CD8 | 1.1182 | 1.1527 | 0.30407 | 0.912 |
| CD4+CD8+T/T | CD3+CD4+CD8+/CD3+ | 1.0455 | 1.1491 | 0.28321 | 0.722 |
| CD4-CD8-T/T | CD3+CD4-CD8-/CD3+ | 7.9055 | 6.64 | 1.20301 | 0.318 |
| Treg/CD4+T | CD3+CD4+CD25bri+CD127-/CD4+ | 8.5345 | 7.8955 | 0.89908 | 0.493 |
| rTreg/CD4+T | CD45RA+CD3+CD4+CD25bri+CD127-/CD4+ | 1.8527 | 1.5482 | 0.27868 | 0.3 |
| mTreg/CD4+T | CD45RA-CD3+CD4+CD25bri+CD127-/CD4+ | 6.6755 | 6.3482 | 0.90285 | 0.725 |
| Texh/T | PD1+TIM3+CD3+/CD3+ | 2.9855 | 3.8527 | 2.10979 | 0.69 |
| CD8+Texh/T | PD1+TIM3+CD3+CD8+/CD3+ | 2.5209 | 2.4682 | 1.61349 | 0.975 |
| CD4+Texh/T | PD1+TIM3+CD3+CD4+/CD3+ | 0.3255 | 1.1064 | 0.67949 | 0.277 |
| PD1+T/T | CD3+PD1+/CD3+ | 28.6009 | 21.6673 | 6.42368 | 0.306 |
| CD8+PD1+T/T | CD3+CD8+PD1+/CD3+ | 15.1827 | 8.4055 | 2.37012 | 0.017** |
| CD4+PD1+T/T | CD3+CD4+PD1+/CD3+ | 11.8273 | 11.9227 | 4.27175 | 0.983 |
| TIM3+T/T | TIM3+CD3+/CD3+ | 17.52 | 16.5591 | 7.66682 | 0.903 |
| CD8+Tim3+T/T | TIM3+CD3+CD8+/T | 11.0427 | 12.61 | 4.06894 | 0.708 |
| CD4+Tim3+T/T | TIM3+CD3+CD4+/CD3+ | 5.4745 | 2.96 | 4.29889 | 0.572 |
| NaiveCD8+T/T | CD27+CD45RA+CCR7+CD8+CD3+/CD3+ | 7.2045 | 8.5364 | 1.49731 | 0.395 |
| CD8+Tcm/T | CD45RA-CCR7+CD27+CD8+CD3+/CD3+ | 2.1909 | 2.0345 | 0.38384 | 0.692 |
| CD8+Tem/T | CD45RA-CCR7-CD8+CD3+/CD3+ | 12.5673 | 11.0636 | 1.70061 | 0.397 |
| TEMRA/T | CD8+CD27-CD45RA+CCR7-CD3+/CD3+ | 20.48 | 20.0118 | 4.51734 | 0.92 |
| CD8+Teff/T | CD45RA+CCR7-CD8+CD3+/CD3+ | 28.426 | 28.354 | 5.84677 | 0.99 |
| NaiveCD4+T/T | CD45RA+CCR7+CD4+CD3+/CD3+ | 22.93 | 16.49 | 5.81787 | 0.294 |
| CD4+Tcm/T | CD45RA-CCR7+CD4+CD3+/CD3+ | 18.3573 | 18.1709 | 4.00791 | 0.964 |
| CD4+Tem/T | CD45RA-CCR7-CD4+CD3+/CD3+ | 9.9209 | 10.9191 | 1.70531 | 0.571 |
| CD4+Teff/T | CD45RA+CCR7-CD4+CD3+/CD3+ | 1.177 | 0.951 | 0.24088 | 0.373 |
| NaiveCD8+T/CD8+Tcm | CD27+CD45RA+CCR7+CD8+CD3+/ CD45RA-CCR7+CD27+CD8+CD3+ | 3.6036 | 15.3373 | 10.37908 | 0.285 |
| NaiveCD8+T/CD8+Tem | CD27+CD45RA+CCR7+CD8+CD3+/ CD45RA-CCR7-CD8+CD3+/CD3+ | 0.6418 | 41.5309 | 40.77661 | 0.34 |
| CD8+Tcm/CD8+Tem | CD45RA-CCR7+CD27+CD8+CD3+/ CD45RA-CCR7-CD8+CD3+ | 0.1909 | 0.5418 | 0.32951 | 0.312 |
| NaiveCD4+T/CD4+Tcm | CD45RA+CCR7+CD4+CD3+/CD45RA-CCR7+CD4+CD3+ | 0.7209 | 10.7391 | 9.92086 | 0.336 |
| NaiveCD4+T/CD4+Tem | CD45RA+CCR7+CD4+CD3+/CD45RA-CCR7-CD4+CD3+ | 1.3227 | 51.1418 | 49.65838 | 0.339 |
| CD4+Tcm/CD4+Tem | CD45RA-CCR7+CD4+CD3+/CD45RA-CCR7-CD4+CD3+ | 2.0518 | 2.0664 | 0.34631 | 0.967 |
| NK/Lym | CD3-CD56+/Lym | 15.4218 | 7.03 | 3.90747 | 0.057 |
| CD56dimCD16+NK/NK | CD56dimCD16+CD3-/CD3-CD56+ | 72.1482 | 68.59 | 7.89222 | 0.662 |
| CD56hiCD16-NK/NK | CD56hiCD16-CD3-/CD3-CD56+ | 3.6173 | 7.1409 | 4.7452 | 0.475 |
| NKT/Lym | CD3+CD56+/Lym | 7.0382 | 7.03 | 1.37042 | 0.995 |
| Lym/WBC | CD45st+/WBC | 12.8427 | 20.3255 | 7.97993 | 0.37 |
| Granulocyte/WBC | CD45dimCD16+SSC++/WBC | 72.9755 | 57.5064 | 6.92307 | 0.049** |
| Eosinophil/WBC | CD45+CD16-SSC++/WBC | 1.1918 | 1.9027 | 0.58529 | 0.252 |
| Basophil/WBC | HLADR-CD123+/WBC | 0.4527 | 0.5591 | 0.10695 | 0.343 |
| pDC/WBC | HLADR+CD123+/WBC | 0.0791 | 0.2573 | 0.17442 | 0.331 |
| PC/WBC | CD38st+CD138+/WBC | 0.0918 | 0.0055 | 0.09145 | 0.367 |
| Monocyte/WBC | CD14+/-CD45+SSC+CD4+/WBC | 8.7891 | 12.7209 | 1.54834 | 0.029** |
| CD14+CD16-Monocyte/Monocyte | CD14+CD16-CD45+SSC+CD4+/CD14+/-CD45+SSC+CD4+ | 61.29 | 63.7318 | 9.88614 | 0.81 |
| CD14+CD16dimMono/Monocyte | CD14+CD16dimCD45+SSC+CD4+/CD14+/-CD45+SSC+CD4+ | 35.5455 | 33.7245 | 9.01849 | 0.844 |
| CD14lowCD16hiMono/Monocyte | CD14-CD16hiCD45+SSC+CD4+/CD14+/-CD45+SSC+CD4+ | 2.5355 | 1.9918 | 1.29972 | 0.685 |
