# Supplementary material for: Hyper-fractionated radiotherapy as a bridging strategy to enhance CAR-T efficacy by regulating T-cell co-stimulatory molecules in relapsed/refractory diffuse large B-cell lymphoma
Source: Front Immunol. 2024 Dec 2;15:1481080. doi: 10.3389/fimmu.2024.1481080 (PMC11646978; doi:10.3389/fimmu.2024.1481080)
Supplement: Supplementary file 3 [file Table3.docx]

**Supplementary Table 3: comparison of the absolute number of immune cell subsets before and after hyper-fractionated radiotherapy analyzed by pared-T test.**

| **cell subsets** | **Phenotype** | **before radiotherapy(×10^9^)** | **after radiotherapy(×10^9^)** | **SD of average changes** | **P value** |
| --- | --- | --- | --- | --- | --- |
| T | CD3+ | 0.6035 | 0.5206 | 0.517 | 0.574 |
| CD4+T | CD3+CD4+ | 0.2087 | 0.1918 | 0.145 | 0.682 |
| CD8+T | CD3+CD8+ | 0.3504 | 0.2845 | 0.387 | 0.551 |
| CD4+CD8+T | CD3+CD4+CD8+ | 0.0058 | 0.0051 | 0.007 | 0.744 |
| CD4-CD8-T | CD3+CD4-CD8- | 0.0385 | 0.0391 | 0.043 | 0.959 |
| Treg | CD3+CD4+CD25bri+CD127- | 0.0157 | 0.0109 | 0.007 | 0.027** |
| rTreg | CD45RA+ CD3+CD4+CD25bri+CD127- | 0.0041 | 0.0027 | 0.003 | 0.095 |
| mTreg | CD45RA- CD3+CD4+CD25bri+CD127- | 0.0115 | 0.0082 | 0.005 | 0.025** |
| Texh | PD1+TIM3+CD3+ | 0.0213 | 0.0161 | 0.044 | 0.678 |
| CD8+Texh | PD1+TIM3+CD3+CD8+ | 0.0189 | 0.0115 | 0.039 | 0.511 |
| CD4+Texh | PD1+TIM3+CD3+CD4+ | 0.0017 | 0.0035 | 0.008 | 0.442 |
| PD1+T | CD3+PD1+ | 0.1907 | 0.1005 | 0.187 | 0.108 |
| CD8+PD1+T | CD3+CD8+PD1+ | 0.1202 | 0.0459 | 0.145 | 0.090 |
| CD4+PD1+T | CD3+CD4+PD1+ | 0.0613 | 0.0480 | 0.052 | 0.379 |
| TIM3+T | TIM3+CD3+ | 0.0820 | 0.0995 | 0.121 | 0.612 |
| CD8+Tim3+T | TIM3+CD3+CD8+ | 0.0603 | 0.0822 | 0.121 | 0.528 |
| CD4+Tim3+T | TIM3+CD3+CD4+ | 0.0177 | 0.0142 | 0.044 | 0.780 |
| NaiveCD8+T | CD27+CD45RA+CCR7+CD8+CD3+ | 0.0459 | 0.0462 | 0.064 | 0.989 |
| CD8+Tcm | CD45RA-CCR7+CD27+CD8+CD3+ | 0.0199 | 0.0098 | 0.028 | 0.219 |
| CD8+Tem | CD45RA-CCR7-CD8+CD3+ | 0.0768 | 0.0614 | 0.072 | 0.454 |
| TEMRA | CD8+CD27-CD45RA+CCR7-CD3+ | 0.1454 | 0.1347 | 0.231 | 0.870 |
| CD8+Teff | CD45RA+CCR7-CD8+CD3+ | 0.1851 | 0.1477 | 0.259 | 0.627 |
| NaiveCD4+T | CD45RA+CCR7+CD4+CD3+ | 0.1384 | 0.0757 | 0.184 | 0.242 |
| CD4+Tcm | CD45RA-CCR7+CD4+CD3+ | 0.0870 | 0.0728 | 0.064 | 0.439 |
| CD4+Tem | CD45RA-CCR7-CD4+CD3+ | 0.0529 | 0.0445 | 0.030 | 0.334 |
| CD4+Teff | CD45RA+CCR7-CD4+CD3+ | 0.0081 | 0.0047 | 0.011 | 0.311 |
| NK | CD3-CD56+ | 0.0948 | 0.0926 | 0.194 | 0.969 |
| CD56dimCD16+NK | CD56dimCD16+CD3- | 0.0695 | 0.0803 | 0.177 | 0.830 |
| CD56hiCD16-NK | CD56hiCD16-CD3- | 0.0029 | 0.0024 | 0.007 | 0.802 |
| NKT | CD3+CD56+ | 0.0588 | 0.0504 | 0.070 | 0.673 |
| Lym | CD45st+ | 0.6945 | 0.7973 | 0.968 | 0.708 |
| Granulocyte | CD45dimCD16+SSC++ | 3.9721 | 2.7763 | 2.102 | 0.063 |
| Eosinophil | CD45+CD16-SSC++ | 0.0858 | 0.0605 | 0.099 | 0.374 |
| Basophil | HLADR-CD123+ | 0.0286 | 0.0178 | 0.026 | 0.156 |
| pDC | HLADR+CD123+ | 0.0041 | 0.0082 | 0.020 | 0.471 |
| PC | CD38st+CD138+ | 0.0058 | 0.0002 | 0.021 | 0.349 |
| Monocyte | CD14+/-CD45+SSC+CD4+ | 0.5586 | 0.4784 | 0.348 | 0.423 |
| CD14+CD16-Monocyte | CD14+CD16-CD45+SSC+CD4+ | 0.3867 | 0.2808 | 0.282 | 0.201 |
| CD14+CD16dimMono | CD14+CD16dimCD45+SSC+CD4+ | 0.1517 | 0.1795 | 0.212 | 0.644 |
| CD14lowCD16hiMono | CD14-CD16hiCD45+SSC+CD4+ | 0.0164 | 0.0132 | 0.016 | 0.502 |
